# Early Versus Late Tracheostomy in Traumatic Spinal Injury: A Narrative Review

**DOI:** 10.3390/neurolint18050085

**Published:** 2026-04-30

**Authors:** Saeed Mahmood, Mohammad Asim, Ayman El-Menyar, Sandro Rizoli, Hassan Al-Thani

**Affiliations:** 1 Department of Trauma Surgery, Hamad Medical Corporation, Doha 3050, Qatar; saeedagm@gmail.com (S.M.); masim1@hamad.qa (M.A.); srizoli@hamad.qa (S.R.); althanih@hotmail.com (H.A.-T.); 2 Department of Clinical Medicine, Weill Cornell Medical School, Doha 24144, Qatar

**Keywords:** traumatic spinal cord injury, early and late tracheostomy, complications, outcomes

## Abstract

Traumatic spinal cord injury (TSCI) frequently necessitates prolonged ventilatory support, raising the clinical dilemma of early versus late tracheostomy. Despite decades of debate, no randomized controlled trials (RCTs) have been conducted exclusively in TSCI populations, and evidence remains largely observational. This review synthesizes contemporary evidence on the timing and outcomes of tracheostomy in acute TSCI. Across multiple cohort studies and meta-analyses, early tracheostomy (≤7 days) is consistently associated with shorter mechanical ventilation duration, shorter ICU length of stay, reduced sedation exposure, and fewer immobility-related complications. Data suggested a lower incidence of ventilator-associated pneumonia, though mortality outcomes remain unchanged. Importantly, cervical-level injuries appear to derive the most significant benefit, while variability in defining “early” versus “late” complicates direct comparisons. Despite methodological limitations, including reliance on retrospective data, inconsistent definitions, and lack of long-term follow-up, cumulative evidence indicates that early tracheostomy improves short-term outcomes. The optimal timing of tracheostomy in TSCI remains uncertain. Current observational evidence suggests that early tracheostomy in cervical SCI is associated with a reduction in the duration of mechanical ventilation, ICU stay, and respiratory complications. These benefits might come from better access to the airways, less anatomical dead space, better clearance of secretions, less need for sedation, together with earlier mobilization and rehabilitation. Mortality outcomes remain inconclusive. In the absence of randomized trials and long-term data, individualized decisions based on injury level, clinical course, and institutional expertise are essential.

## 1. Introduction

Traumatic spinal cord injury (TSCI), especially at the cervical level, often causes severe respiratory compromise, requiring endotracheal intubation and ventilatory support [1]. High cervical spinal cord injury (CSCI) disrupts neural pathways to the diaphragm and accessory respiratory muscles, drastically reducing ventilatory capacity [2]. Loss of sympathetic innervation increases bronchial smooth muscle tone and mucous production [3]. When prolonged ventilation is expected, tracheostomy is generally preferred over extended trans-laryngeal intubation for easier weaning and pulmonary care [4]. Notably, 81–91% of patients with complete cervical SCI require mechanical ventilation [5], while about a quarter of those with incomplete SCI need support beyond 48 h [6]. Many patients requiring mechanical ventilation eventually undergo tracheostomy for long-term airway management. Tracheostomy offers several advantages over prolonged endotracheal intubation in CSCI: improved ventilation, reduced anatomical dead space, and better clearance of bronchial secretions [7]. It also enables earlier sedation reduction, facilitates speech and swallowing, and allows safer oral intake, improving overall patient management and comfort [8]. However, as an invasive procedure, it can cause airway stenosis and other complications.

The timing of tracheostomy in critically ill patients has been debated for decades. Guidelines suggest tracheostomy if mechanical ventilation is expected for 7–10 days or more, as prolonged trans-laryngeal intubation increases the risk of complications [9]. However, no conclusive evidence exists to define the optimal timing. Thus, decisions must be individualized, considering risks and benefits for patients who are likely to require extended intubation [10]. Notably, tendencies and preferences often guide the decision between early and late tracheostomy. Some emergency clinicians favor early tracheostomy to reduce complications and speed weaning [11].

Others prefer a cautious approach to avoid unnecessary procedures if extubation is possible without tracheostomy [12]. An earlier study suggested that early tracheostomy reduces mechanical ventilation in CSCI patients, regardless of neurological involvement [13]. Still, there is no consensus on specific thresholds for early versus late tracheostomy. In CSCI, the decision remains particularly complex.

We undertook this narrative review to synthesize current knowledge on early versus late tracheostomy in acute TSCI, covering epidemiology, pathophysiology, management strategies, and clinical outcomes. This review synthesizes contemporary evidence (inception to January 2026) on the timing and outcomes of tracheostomy in acute TSCI using Google scholar and PUBMED. The literature was reviewed by three authors (SM, MA and AE). We included all relevant articles published in the English language.

## 2. Epidemiology of Traumatic Spinal Cord Injuries

TSCI is a major cause of mortality and long-term disability worldwide, with substantial socioeconomic impact [14]. Global incidence estimates of TSCI range from 8 to 246 cases per million annually, reflecting marked geographic variation in injury patterns and healthcare systems [15]. Falls and motor vehicle Crashes (MVCs) remain the leading mechanisms of injury, while the average age at injury has increased in many regions [16,17,18]. CSCI represents the subgroup with the greatest risk of respiratory failure, as about 75% of patients may require endotracheal intubation, and 11–35% subsequently undergo tracheostomy for prolonged airway and pulmonary care [10,19]. These epidemiological data underscore the clinical significance optimizing tracheostomy timing in this high-risk population [20].

### 2.1. Pathophysiology

Acute TSCI is often associated with alterations in cardiopulmonary function that require management in the ICU. The respiratory consequences are most pronounced in cervical injuries, where disruption of diaphragmatic, intercostal, and abdominal muscle innervation can markedly impair ventilation, cough effectiveness, and secretion clearance. Injuries above C3–C5 may cause severe diaphragmatic weakness or apnea, whereas lower cervical lesions may preserve partial diaphragmatic function but still compromise respiratory mechanics [21]. In the acute phase, spinal shock and chest wall muscle causes paralysis reduce vital capacity and promote shallow, inefficient breathing, predisposing patients to atelectasis, mucus plugging, and pneumonia [22,23]. As a result, patients develop shallow, rapid breathing that is inefficient and predisposes them to microatelectasis, mucus plugging, and pneumonia [23]. In addition, disruption of sympathetic pathways may produce neurogenic shock with bradycardia, hypotension, and vasodilation, particularly in high cervical and upper thoracic injuries [24,25]. The level and severity of injury therefore remain key determinants of respiratory failure, ventilator dependence, and the potential need for tracheostomy [2,26,27]. These pathophysiological mechanisms explain why patients with severe CSCI may particularly benefit from timely airway management and individualized decisions regarding tracheostomy timing.

### 2.2. Respiratory Complications and the Role of Mechanical Ventilation

Respiratory complications are a leading cause of morbidity and mortality in patients with acute SCI, with pulmonary issues, especially pneumonia, accounting for a large proportion of early deaths [28]. Weak cough, impaired secretion clearance, and reduced lung volumes predispose patients to secretion retention, atelectasis, and pneumonia. In the ICU setting, ventilator-associated pneumonia (VAP) remains a significant concern, with risk increasing during prolonged endotracheal intubation [29,30,31]. Delayed/missed diagnosis and treatment of pulmonary infection may adversely affect recovery and prolong ICU stay [28]. Mechanical ventilation is often lifesaving in acute CSCI, but prolonged trans-laryngeal intubation (>7–10 days) can cause laryngeal injury, tracheal mucosal damage, and granuloma formation, and long-term airway stenosis [32]. Additionally, an endotracheal tube necessitates deep sedation, delayed immobilization, impairs communication, psychological well-being and may hinder rehabilitation [33]. In contrast, early tracheostomy may reduce airway resistance, improve secretion management, and facilitate lighter sedation and progressive weaning [34]. Accordingly, the timing of tracheostomy requires balancing the risks of prolonged intubation against the benefits and procedural risks of earlier tracheostomy.

### 2.3. Tracheostomy Procedure

Tracheostomy is a common airway procedure performed to provide prolonged ventilatory support, facilitate weaning, and improve airway access in critically ill patients [4]. It is generally performed using either an open surgical technique or a percutaneous dilatational approach [35]. The choice is influenced by patient anatomy, cervical fixation, local expertise, and resource availability [4]. Both techniques are widely used in SCI populations and can be performed safely when appropriate precautions are taken. In patients with cervical spinal cord injury, the key clinical issue is often not the technique itself, but the optimal timing of the procedure in relation to anticipated ventilator dependence, pulmonary complications, and rehabilitation goals [11]. Concerns such as recent anterior cervical surgery, neck immobilization, coagulopathy, or difficult anatomy may affect procedural planning [36], but these factors should be weighed against the risks of prolonged translaryngeal intubation.

#### 2.3.1. Indications for Tracheostomy

Tracheostomy is considered in patients with CSCI when prolonged airway or ventilatory support (>7–10 days) is anticipated [13]. Common indications include prolonged ventilatory dependence failed extubation or difficult weaning, ineffective cough with secretion retention, recurrent atelectasis or pneumonia, impaired airway protection, and the expectation of extended critical care needs [37]. The likelihood of tracheostomy is higher in patients with complete injuries, high cervical lesions, severe neurological impairment, and associated thoracic trauma [4]. Therefore, indications for tracheostomy are closely linked not only to the need for the procedure itself, but also to determining the most appropriate timing.

#### 2.3.2. Complications of Tracheostomy

Although tracheostomy is generally safe, it remains an invasive procedure with potential early and late complications. Early complications may include bleeding, pneumothorax, pneumomediastinum, local infection, tube malposition and subcutaneous emphysema [38]. Many early complications are preventable or manageable with experienced operators and proper post-procedure care [38]. Late complications include tracheal stenosis or tracheomalacia, tracheo-esophageal fistula, and granulation tissue formation at the stoma, which may require surgical revision [39]. In patients with cervical spinal cord injury, additional concerns may include challenging neck access due to immobilization or recent cervical fixation [4]. However, contemporary studies suggest that when performed with appropriate technique and patient selection, tracheostomy-related complication rates are low and should be weighed against the risks of prolonged endotracheal intubation [11]. Therefore, the decision is not simply whether complications can occur, but whether earlier tracheostomy offers a more favorable overall risk–benefit profile than delayed intervention.

#### 2.3.3. Early vs. Late Tracheostomy

The optimal timing of tracheostomy in CSCI remains debated, largely because definitions of “early” and “late” vary across studies. Most published studies define early tracheostomy as within 4–7 days of intubation or injury, although some studies use cut-offs ranging from 3 to 14 days [40,41]. Late tracheostomy usually refers to any tracheostomy performed after the early window, or to continued endotracheal tube management without a tracheostomy. The lack of a uniform definition makes it difficult to directly compare studies [40]. Despite this heterogeneity, the overall evidence trend favors earlier intervention in appropriately selected patients. A landmark prospective randomized trial by Rumbak et al. [42] involving 120 critically ill medical ICU patients demonstrated marked benefits of early tracheostomy (<48 h) compared with delayed tracheostomy (around 2 weeks). The authors found that the early tracheostomy group had significantly lower mortality (31.7% vs. 61.7%), lower incidence of pneumonia, and fewer unplanned extubation, along with dramatically shorter ICU stays and ventilation durations than the prolonged intubation group. This suggested that, at least in selected patients, early tracheotomy can improve clinical outcomes and reduce complications of prolonged intubation. However, a meta-analysis of 9 randomized trials (2072 ICU patients) reported that early tracheostomy (<1 week) did not confer a significant difference in key outcomes, including short-term or long-term mortality, ICU length of stay, duration of mechanical ventilation, or VAP incidence compared to late tracheostomy or prolonged intubation [12]. This suggests that in unselected ICU populations, early tracheostomy may not universally improve outcomes, possibly due to heterogeneity in patient conditions. Also, Hosokawa et al. [43] reviewed 12 randomized controlled trials (RCTs) involving 2689 patients and reported that early tracheostomy increased ventilator-free days, shortened ICU stay and sedation time, and modestly reduced long-term mortality, without increasing complications. These findings suggest efficiency and outcome benefits with earlier intervention. A meta-analysis of 15 RCTs (3003 patients) by Deng et al. [44] showed that early tracheostomy reduced ICU length of stay and ventilation duration, but did not improve short-term mortality, pneumonia rates, or overall hospital stay. The authors concluded that early tracheostomy primarily enhances ICU efficiency rather than survival. Therefore, across ICU populations, early tracheostomy consistently improves ICU efficiency (shorter ventilation time, shorter ICU stay, and reduced sedation), but its effects on mortality and infection remain inconclusive.

Table 1 summarizes key observational studies comparing early versus late tracheostomy in patients with SCI [7,9,13,40,45,46,47,48,49,50,51,52,53,54,55,56,57,58,59]. Patients with acute CSCI represent a distinct subgroup, as they almost invariably require prolonged mechanical ventilation due to neurological impairment and may therefore derive particular benefit from early tracheostomy. Ethical and logistical challenges limit the feasibility of RCTs in this cohort; thus, current evidence is primarily derived from observational studies and systematic reviews.

A recent multicenter analysis of 1592 patients with acute CSCI across 358 trauma centers demonstrated that early tracheostomy significantly reduced major complications, immobility-related events, and hospital, ICU, and ventilation durations. Importantly, these benefits were consistent across surgical approaches (anterior, posterior, or combined) [57].

Similarly, Romero et al. [45] reported that early tracheostomy (<7 days) was associated with shorter ICU and hospital stays and a reduced incidence of VAP, although mortality remained unchanged. Another study from Spain confirmed the safety of early tracheostomy, whether surgical or percutaneous, and underscored its role in lowering complications and optimizing resource utilization [46]. Choi et al. [9] and Guirgis et al. [13] further supported these findings, noting reductions in ICU stay, ventilation duration, and ICU mortality, particularly in high cervical injuries. Earlier studies from Korea [47] and the United States [48] corroborated these findings, showing improved respiratory outcomes and shorter ICU stays without increased infection or mortality. Consistently, Lozano et al. [41] reported lower pneumonia rates and reduced healthcare resource utilization in early tracheostomy patients in a large Spanish cohort.

Data from Asian cohorts align with these findings. Wang et al. [7] and Shan et al. [51] demonstrated that early or immediate tracheostomy after cervical fixation reduced ventilation duration and hospitalization, without increasing the risk of surgical site infection or mortality. Complementary evidence from ACS-TQIP database analyses by Anand et al. [49] and Khan et al. [50] showed consistent reductions in respiratory complications, VAP, and ICU/hospital stay, though mortality benefits remained limited.

More recent investigations have offered additional insights. Wang et al. [52] showed that both early (≤4 days) and intermediate (4–10 days) tracheostomies were associated with better functional recovery and lower mortality than delayed procedures. Sun et al. [53] further introduced the strategy of intraoperative one-stage tracheostomy, which proved effective in reducing pulmonary infections, ventilation days, ICU and hospital stay, and overall healthcare costs. In contrast, Kelly et al. [55] observed that delaying tracheostomy after cervical fixation prolonged ICU and hospital stay without reducing infection risk. Large multicenter investigations have confirmed these trends. Studies by Balas et al. [56] and Essa et al. [57] demonstrated that early tracheostomy consistently reduced complication rates, shortened ICU stay and shortened mechanical ventilation duration. Importantly, these benefits were observed regardless of the surgical approach, highlighting the robustness of the evidence supporting early intervention.

### 2.4. Synthesis of Evidence from Systematic Reviews and Meta-Analyses

Recent systematic reviews and meta-analyses have also synthesized these findings. Mubashir et al. [58] reviewed eight studies involving 1220 patients with acute traumatic SCI. Early tracheostomy, generally defined as within seven days of injury or intubation, was associated with markedly shorter ICU stay and reduced ventilation duration. At the same time, overall mortality was lower in the early group, but this difference did not reach statistical significance. Subgroup analyses, however, suggested survival benefits in patients with higher injury severity. Pneumonia rates were comparable between groups. The authors concluded that early tracheostomy improves ICU resource utilization and may enhance survival in selected subgroups, while stressing the need for higher-quality prospective studies.

Foran et al. [11] expanded the evidence base by analyzing 17 studies involving 2804 patients with acute cervical or high thoracic SCI. Their findings corroborated those of Mubashir et al. [58], showing no mortality benefit with early tracheostomy but substantial improvements in resource-related outcomes. Specifically, early tracheostomy reduced the duration of mechanical ventilation by 13.1 days, ICU stay by 10.2 days, and total hospital stay by 7.4 days. Notably, this analysis also demonstrated a lower incidence of ventilator-associated pneumonia and fewer tracheostomy-related complications in the early group, suggesting that early intervention may not only enhance efficiency but also mitigate risks. The authors concluded that while the optimal timing remains uncertain due to limited randomized evidence, early tracheostomy provides significant clinical and logistical benefits.

A recent review by Zameni et al. [59] in 2025, including 17 studies and 3853 patients, further reinforced these conclusions. Early tracheostomy (≤7 days) was consistently associated with shorter ventilation, ICU, and hospital stays, as well as significant reductions in pneumonia and tracheostomy-related complications. Mortality remained unaffected, but the larger pooled dataset provided more robust evidence of clinical and resource-use benefits. The authors advocated considering early tracheostomy in appropriate SCI patients to streamline ICU care and improve outcomes.

The most recent systematic review and meta-analysis by Coffin et al. [60] including 14 studies and 693 CSCI patients who underwent anterior cervical decompression further contributed to the contemporary evidence. Their findings were consistent with prior meta-analyses, demonstrating no significant survival benefit with early tracheostomy, while showing meaningful improvements in resource-related outcomes. Specifically, early tracheostomy was associated with shorter duration of mechanical ventilation, reduced ICU length of stay, and shorter overall hospital stay. Importantly, the analysis also found no increased risk of postoperative wound infection following anterior cervical surgery. The authors concluded that, in appropriately selected patients, early tracheostomy after anterior cervical decompression appears safe and offers important clinical and logistical advantages.

Across these observational studies and systematic reviews, the evidence consistently demonstrates that early tracheostomy reduces ventilator days and ICU/hospital stays, often by more than a week, an outcome of clear clinical significance. For instance, early tracheostomy shortened the duration of mechanical ventilation by a median of 13 days, ICU stay by 10 days, and total hospital stay by 7 days compared to late tracheostomy in recent meta-analyses and cohort studies. Presenting these absolute reductions in days provides a tangible sense of the clinical benefits that can be expected. The impact on pneumonia and VAP has been more variable in earlier literature, but more recent SCI-focused studies indicate early tracheostomy can indeed lower pneumonia incidence. Early tracheostomy likely enhances pulmonary clearance and allows earlier reduction in sedation, which, in turn, may reduce the risk of ventilator-associated infections.

Mortality has not been definitively improved with early tracheostomy in any meta-analysis, as none has shown a statistically significant mortality benefit [11,59]. However, one analysis noted a significant survival benefit in a certain subgroup of early-tracheostomy patients [58]. It is possible that mortality in acute SCI is driven more by the severity of the neurologic injury and concomitant trauma rather than by tracheostomy timing, and the sample sizes thus far are underpowered to detect a mortality difference. Nevertheless, it’s reassuring that early tracheostomy appears not to worsen mortality. Importantly, no study has reported worse outcomes with early tracheostomy; at minimum, outcomes are equivalent, and at best, significantly improved.

It is worth noting that definitions of “early” versus “late” vary across studies. While most studies used a seven-day cut-off, some extended “early” to within ten days. Regardless, outcomes were consistent, showing that tracheostomy performed within the first week to ten days was associated with fewer complications and shorter ICU and hospital stays compared with later interventions. Thus, the cumulative evidence strongly supports early tracheostomy—defined pragmatically within the first week after injury—as an effective strategy for improving short-term outcomes in acute CSCI patients.

### 2.5. Clinical Decision-Making Considerations and Recommendations in CSCI Patients

The rationale for tracheostomy timing in cervical spinal cord injury is grounded in the underlying respiratory physiology and risk of pulmonary complications. High cervical injuries can impair diaphragmatic, intercostal, and abdominal muscle function, resulting in weak cough, secretion retention, atelectasis, and prolonged ventilator dependence [21]. In this context, tracheostomy may provide more secure airway access, improve secretion clearance, reduce airway resistance and anatomical dead space, and permit lighter sedation with earlier patient interaction [61]. However, the procedure remains invasive and carries potential risks, including bleeding, infection, tracheal injury, and late airway stenosis. Therefore, the choice between early and delayed tracheostomy requires balancing the anticipated benefits of earlier respiratory support and rehabilitation [62] against the possibility of avoiding an unnecessary procedure in patients likely to recover sufficient spontaneous ventilation.

Deciding when to perform a tracheostomy in a patient with traumatic CSCI requires an individualized assessment that considers injury-related factors, patient factors, and institutional or provider practices [63]. High cervical injuries, complete cord syndromes, and patients with early evidence of inability to wean are strong candidates for early tracheostomy [11,13,29,47]. Studies have highlighted several factors that predict the need for tracheostomy in CSCI. These include a markedly reduced American Spinal Injury Association (ASIA) motor score, indicating profound paralysis; higher-level injuries, particularly above C4; the presence of paradoxical breathing patterns; and excessive airway secretions, all of which are strongly associated with subsequent tracheostomy requirements [47,50,64]. One retrospective analysis found that if a CSCI patient remained intubated for more than 4 days post-injury and failed weaning, then proceeding with early tracheostomy was beneficial and shortened the ICU stay [41]. On the other hand, patients with incomplete lower cervical injuries (e.g., C5–C7) who have some preserved diaphragmatic function might be given a trial of extubation or further weaning attempts before committing to tracheostomy, especially if they show signs of recovery [65]. New diaphragmatic pacing technologies, such as phrenic nerve or diaphragm pacers, may allow some patients with high SCI to breathe without a ventilator; however, they require time and specialized evaluation and cannot replace the need for initial airway management [66].

The decision regarding tracheostomy timing is influenced by a combination of clinical, prognostic, and organizational factors [67,68]. Clinically, early tracheostomy is more strongly considered in patients with high cervical complete injuries, persistent ventilator dependence, ineffective cough, excessive secretions, recurrent atelectasis, or failed spontaneous breathing and extubation attempts [9]. In such cases, earlier airway access may reduce sedation exposure, facilitate airway clearance, improve communication, and accelerate mobilization and rehabilitation [69]. Conversely, delayed tracheostomy may be reasonable in patients with incomplete or lower cervical injuries who demonstrate improving respiratory mechanics and a realistic likelihood of early extubation, thereby avoiding an unnecessary invasive procedure [11]. Beyond patient factors, organizational determinants also play an important role. ICU bed pressures, availability of experienced operators, access to bronchoscopy or operating theater resources, respiratory therapy support, rehabilitation pathways, and institution-specific protocols may all influence the timing of intervention [62]. Variability in these logistical factors likely explains part of the heterogeneity observed across published studies and highlights that tracheostomy timing is not solely a procedural decision, but a broader system-based clinical strategy.

A stepwise decision algorithm can be adopted. First, if the patient has a complete injury at C1–C3 and is ventilator-dependent, proceed with tracheostomy within days 3–5 post-injury. Second, if the patient has a complete injury at C4 or a complete high cervical injury with early signs of weaning difficulty (such as failed extubation attempts or persistent need for ventilatory support by day 4), consider tracheostomy by day 7. For patients with incomplete injuries (C5–C7) or preserved diaphragmatic function, continue monitoring and attempt weaning or extubation. If extubation is not successful by day 7 or ventilator dependence persists, reassess for tracheostomy. Additional patient factors, such as excessive secretions, paradoxical breathing, or markedly reduced ASIA motor scores, may prompt earlier tracheostomy regardless of exact injury level. Consider institutional resources and practitioner experience that prioritize patient safety and successful early weaning.

Moreover, there remains substantial variation in practice regarding the timing of tracheostomy. Some trauma centers have protocols to perform tracheostomy early (day 3–5) for all high cervical injuries, while others prefer to wait and see if the patient can be weaned off the endotracheal tube [45,65,70]. These institutional and clinician preference differences can confound direct comparisons of outcomes, as noted in prior reviews. In centers where early tracheostomy is routinely practiced, the entire care pathway is often designed to leverage its advantages, facilitating earlier mobilization, more intensive physiotherapy, and streamlined respiratory care [71]. Conversely, in centers that favor delayed tracheostomy, clinical management may instead prioritize repeated weaning attempts and prolonged trials of extubation readiness [72]. These context-specific strategies can significantly influence outcomes and are difficult to fully account for in retrospective analyses. Such variability underscores the importance of establishing standardized protocols or, ideally, conducting RCTs in high-risk populations.

At present, no formal guidelines exist that are specifically tailored to the management of tracheostomy timing in patients with acute SCI. While some best practice recommendations have supported early tracheostomy, considerable variability remains in its implementation following CSCI [73]. Major international critical care societies offer no definitive guidance; instead, they emphasize individualized decision-making, typically after 7–10 days of endotracheal intubation. This reflects the scarcity of RCTs and the difficulty of isolating SCI patients in prospective studies. However, the synthesis of more recent evidence on acute SCI patients indicates a clear trend toward the benefits of earlier tracheostomy [11,58,59,63,74]. These studies consistently report reductions in mechanical ventilation duration, ICU length of stay, and sedation-related complications. Importantly, these advantages appear more pronounced in CSCI patients. Taken together, the current body of evidence supports a gradual shift in clinical practice toward favoring early tracheostomy in patients with CSCI who are expected to require prolonged ventilatory support. Although specific guidelines are currently unavailable, data indicated that clinicians should lower their threshold for early intervention while carefully considering individualized factors, such as neurological prognosis, comorbidities, and overarching treatment objectives. In the absence of dedicated SCI guidelines, this evidence-informed approach provides a pragmatic, clinically relevant framework for decision-making in practice.

The current evidence underscores the need for high-quality prospective research, ideally multicenter RCTs, to determine the optimal timing of tracheostomy in acute CSCI. Such studies should compare early versus late intervention, evaluating outcomes such as ventilator-free days, VAP incidence, ICU and hospital length of stay, survival, neurological recovery, and patient-centered outcomes, including comfort and quality of life.

It is crucial to proactively involve a multidisciplinary team including speech therapists, rehabilitation specialists, respiratory therapists, nurses, and social workers in the design and execution of future trials. Their expertise can help ensure that study protocols comprehensively address the full spectrum of recovery, such as early communication, swallowing function, weaning readiness, and rehabilitation progress. Broader team inclusion can also improve patient-centered outcome measurement and facilitate holistic post-ICU transitions for patients and families.

Until such evidence is available, clinical practice must rely on observational data and sound judgment. A practical approach is to perform tracheostomy around day 7 in patients who remain ventilator-dependent, while deferring the procedure in those demonstrating recovery and potential for extubation. Although patient and family preferences should be considered, limited decision-making capacity in the acute setting often requires the care team to act in the patient’s best interests. Future work should also aim to establish standardized definitions of “early” versus “late” tracheostomy and develop SCI-specific guidelines. Table 2 compares early and late tracheostomy [75,76,77,78]. Notably, the level of evidence or the consistency of the evidence was not reported in this table as no quantitative or qualitative analysis was made.

### 2.6. Limitations

It is important to acknowledge the limitations of the current evidence. Notably, data are mainly from observational studies, mostly retrospective cohort studies, as no RCTs have been performed exclusively in SCI. Therefore, even meta-analyses aggregate lower-level evidence that is inherently biased. Moreover, unmeasured confounders (such as injury severity, surgeon judgment, etc.) could influence the results. However, the consistency of findings across multiple independent cohorts lends credibility to the observed benefits of early tracheostomy. Another limitation is variability in defining “early” and “late” and differences in cut-off times, though again, the overall trends remained similar across definitions. Also, it is worth noting that some studies combined patients with cervical and thoracic SCI; thoracic SCI patients might not need tracheostomy as often, and this could skew results if not analyzed separately [79,80]. Another key limitation is the absence of long-term outcome data, as none of the available studies extended follow-up beyond hospital discharge. As highlighted by Foran et al. [11], evidence remains insufficient regarding long-term and patient-centered outcomes, including comfort, quality of life, and functional recovery. Moreover, observational cohort data may confound interpretation, including competing risks across multiple outcomes and time bias. Lastly, the evidence from general ICU (pathophysiological context) should be clearly separated from SICU- specific evidence (basis for conclusions). In general, and not specific to ours, narrative reviews (in general) lack the assessment of quality of the included studies.

## 3. Conclusions

The optimal timing of tracheostomy in TSCI remains uncertain. Current observational evidence suggests that early tracheostomy in cervical SCI is associated with a reduction in the duration of mechanical ventilation, ICU stay, and respiratory complications, without increasing risk. Mortality outcomes remain inconclusive, likely due to the predominant influence of neurological injury severity rather than tracheostomy timing. In the absence of randomized trials and long-term data, individualized decisions based on injury level, clinical course, and institutional expertise are essential. Overall, the current body of evidence indicates that early tracheostomy could be a beneficial strategy in acute CSCI, with the potential to improve patient outcomes while optimizing healthcare resource utilization.

## Figures and Tables

**Table 1:** Summary of studies on Early versus Late Tracheostomy in Spinal Cord Injury Patients [7,9,13,40,45,46,47,48,49,50,51,52,53,54,55,56,57,58,59].

Author (Year)	Country	Study Population	Study Design	Early vs. Late Tracheostomy	Complications & Outcomes	Key Findings
Wang et al., 2020 [7]	China	45 CSCI	Retrospective	ET: immediate post-surgery (*n* = 25), LT: 3–12 days post-surgery (*n* = 20)	ET reduced MV duration, indwelling tube days, and hospital stay. No increase in pneumonia, mortality, or incision infection.	ET is safe and effective; it reduces hospital stay without added infection risk.
Choi et al., 2013 [9]	Korea	21 CSCI	Retrospective	ET: 1–10 days (*n* = 10), LT: >10 days (*n* = 11)	ET reduced ICU stay (20.8 vs. 38 days) and mechanical ventilation (5.2 vs. 29.2 days). No significant difference in hospital stays and complications.	ET is beneficial in reducing ICU stay and ventilation duration.
Guirgis et al., 2016 [13]	Oman	69 CSCI	Retrospective	ET: ≤7 days, LT: >7 days	ET reduced ventilation days (9.3 vs. 13.7); high CSCI was associated with lower ICU mortality. No difference in ICU LOS.	Supports ET, especially in high CSCI patients.
Lozano et al., 2018 [41]	USA	98 CSCI	Retrospective	ET: ≤4 days (*n* = 39), LT: ≥5 days (*n* = 59)	There was not statistically significant significant difference in ICU stays, hospital stay, or mortality between groups.	ET after anterior cervical fusion is safe and does not raise infection risk.
Romero et al., 2009 [45]	Spain	152 (121 CSCI; 31 TSCI)	Retrospective	ET: <7 days (*n* = 71), LT: ≥7 days (*n* = 81)	ET reduced ICU stay (36.5 vs. 54.4 days) and mechanical ventilation (26.0 vs. 48.7 days). No difference in the rate of ventilator-associated pneumonia and mortality among the two groups.	ET improves pulmonary outcomes and reduces ICU stay.
Ganuza et al., 2011 [46]	Spain	323 (208 CSCI; 115 TSCI)	Retrospective	ET: ≤7 days (*n* = 101), LT: >7 days (*n* = 114)	ET had favorable effects, reduced complications, and was safe via both surgical & percutaneous techniques. Mortality low.	ET may improve outcomes and reduce resource use; both techniques are safe.
Beom & Seo, 2018 [47]	Korea	49 CSCI	Retrospective	ET: ≤7 days (*n* = 10), LT: >7 days (*n* = 12)	The ET group had a shorter ICU stay (11.4 vs. 19.7 days).	ET should be considered if extubation is not possible within 4 days post-surgery.
Flanagan et al., 2018 [48]	USA	70 CSCI	Retrospective	ET: ≤7 days (*n* = 37), LT: >7 days (*n* = 33)	ET reduced ventilator days (23.9 vs. 36.9), ICU stay (20.7 vs. 26.0), and decannulation (53 vs. 74.3 days). No pneumonia/mortality difference.	ET is safe and associated with improved respiratory outcomes.
Anand et al., 2020 [49]	USA	5980 CSCI	Retrospective	ET: ≤4 days (*n* = 1010), LT: >4 days (*n* = 4970)	ET reduced respiratory complications (30% vs. 46%), more ventilator/ICU-free days, and shorter hospital LOS. No mortality difference.	ET improves respiratory outcomes and reduces LOS.
Khan et al., 2020 [50]	USA	1139 CSCI	Retrospective	ET: ≤7 days (*n* = 280), LT: >7 days (*n* = 859)	ET reduced overall complications (OR 0.57), pneumonia, MV days, ICU & hospital LOS. No mortality difference.	ET improves complications and resource use.
Shan et al., 2020 [51]	China	133 CSCI	Retrospective	ET: ≤4 days post-ACF (*n* = 51), LT: >4 days (*n* = 82)	No difference in surgical site infection. ET reduced ventilation duration. No significant difference in ICU length of stay or mortality.	Early percutaneous tracheostomy after ACF is safe and reduces MV without increasing infection.
Wang et al., 2021 [52]	China	124 CSCI	Retrospective	ET: ≤4 days (*n* = 46), Medium: 4–10 days (*n* = 38), LT: ≥10 days (*n* = 40)	LT is linked to longer MV, ICU stay, higher pneumonia, and ICU mortality. Early/medium improved functional recovery.	Early/medium tracheostomy provides better clinical and functional outcomes.
Sun et al., 2023 [53]	China	41 CSCI	Retrospective	ET: intraoperative one-stage trach (*n* = 10), LT: post-op when required (*n* = 13), no tracheostomy (*n* = 8)	ET reduced pneumonia by 7 days, MV duration, ICU/hospital stay, and costs. Better PaO2 and fewer complications.	One-stage intraoperative tracheostomy is safe and reduces respiratory complications.
Sun et al., 2023 [54]	China	63 CSCI	Retrospective	ET: ≤7 days, LT: >7 days	ET was associated with shorter hospital and ICU stay despite greater injury severity and worse neurological status.	ET improved resource utilization, and clinical predictors may help guide optimal timing and patient selection.
Kelly et al., 2023 [55]	USA	1438 CSCI		ET: <7 days, LT: ≥7 days	No SSI difference (1.6% vs. 1.2%). Late trach is linked to longer ICU/hospital LOS, ventilation, and morbidity.	Delaying tracheostomy after cervical fixation is not beneficial.
Balas et al., 2023 [56]	USA (ACS-TQIP)	2001 CSCI	Retrospective,	ET: ≤7 days, LT: >7 days	ET reduced major/immobility-related complications, ICU LOS (by 8.2 days), and ventilation (by 6.7 days). Variability across centers.	ET was associated with reduced in-hospital complications, time in the ICU, and mechanical ventilation.
Essa et al., 2024 [57]	USA (ACS-TQIP)	1592 CSCI	Retrospective	ET: ≤7 days post-surgery, LT: >7 days	ET reduced complications (OR 0.67), immobility-related complications (OR 0.78), and shorter hospital LOS and ventilation.	ET is associated with reduced complications, LOS, and ventilation time regardless of surgical approach.

ET: early tracheostomy; LT: late tracheostomy; CSCI: Cervical spinal cord injury; LOS: length of stay; ICU: Intensive care unit. MV: Mechanical ventilation; OR: Odd ratio; SSI: Surgical site infection; ACF: anterior cervical fusion; ACS-TQIP: American College of Surgeons Trauma Quality Improvement Program.

**Table 2:** A comparison between early and late tracheostomy refs: [75,76,77,78].

Variable	Early Tracheostomy	Late Tracheostomy
Impact on weaning process	Facilitates weaning process due to decreased airway resistance and dead space.	The endotracheal tube itself can be a barrier to successful spontaneous breathing trials
Duration of Mechanical Ventilation	shorter total and post-procedural ventilation time. Proactive intervention to avoid the complications of prolonged translaryngeal intubation and facilitate weaning.	longer duration of invasive ventilation. Allow for a trial of extubation; avoid an unnecessary procedure if the patient can breathe independently
Hospital Length of Stay	Shorter stays (ICU reduction of ~9–13 days; Hospital reduction of ~7–8 days).	Longer stays in acute care settings.
Ventilator-Associated Pneumonia (VAP)	Associated with a lower incidence of VAP and overall pneumonia.	Higher risk of developing VAP during prolonged intubation.
Mortality	Mixed results. No significant difference in overall mortality, though some studies show a reduction in high cervical (C1–C2) SCI.	Standard mortality outcomes but may be higher in specific high-cervical subgroups due to respiratory failure.
Airway Complications	Reduced risk of tracheal stenosis and granulomas from prolonged orotracheal intubation. Easier secretion management via direct suctioning, potentially reducing ventilator-associated pneumonia (VAP) risk.	Increased risk of laryngotracheal injury, such as subglottic stenosis, due to long-term tube placement. More difficult secretion clearance around the endotracheal tube, potentially increasing VAP risk
Patient Comfort & Management	Facilitates earlier communication (vocalization), oral nutrition, and reduced sedation requirements. Procedure-related risks	Prolonged sedation and delayed mobilization. Higher cumulative risk of complications from prolonged intubation
Resource Utilization	Lower total direct costs and hospital charges.	Higher costs associated with prolonged intensive care and complications

## Data Availability

No new data were created or analyzed in this study. Data sharing is not applicable to this article.

## Abbreviations

ASCI	Acute spinal cord injury
CSI	Cervical cord injury
ETT	Endotracheal tube
ICU	Intensive care unit
PT	Percutaneous tracheostomy
MV	Mechanical ventilation
SCI	Spinal cord injury
TSCI	Traumatic spinal cord injury
